# Epitaxial Lift-Off of Flexible GaN-Based HEMT Arrays with Performances Optimization by the Piezotronic Effect

**DOI:** 10.1007/s40820-021-00589-4

**Published:** 2021-02-10

**Authors:** Xin Chen, Jianqi Dong, Chenguang He, Longfei He, Zhitao Chen, Shuti Li, Kang Zhang, Xingfu Wang, Zhong Lin Wang

**Affiliations:** 1 Laboratory of Nanophotonic Functional Materials and Devices, Institute of Semiconductor Science and Technology, South China Normal University, Guangzhou, 510631 People’s Republic of China; 2 Institute of Semiconductor, Guangdong Academy of Sciences, Guangzhou, 510651 People’s Republic of China; 3 Beijing Institute of Nanoenergy and Nanosystems, Chinese Academy of Sciences, Beijing, 100083 People’s Republic of China; 4 School of Materials Science and Engineering, Georgia Institute of Technology, Atlanta, GA 30332-0245 USA

**Keywords:** AlGaN/AlN/GaN heterojunction, Epitaxial lift-off, Flexible membrane, Two-dimensional electron gas, Piezotronic effect

## Abstract

High-electron-mobility transistors (HEMTs) are a promising device in the field of radio frequency and wireless communication. However, to unlock the full potential of HEMTs, the fabrication of large-size flexible HEMTs is required. Herein, a large-sized (> 2 cm^2^) of AlGaN/AlN/GaN heterostructure-based HEMTs were successfully stripped from sapphire substrate to a flexible polyethylene terephthalate substrate by an electrochemical lift-off technique. The piezotronic effect was then induced to optimize the electron transport performance by modulating/tuning the physical properties of two-dimensional electron gas (2DEG) and phonons. The saturation current of the flexible HEMT is enhanced by 3.15% under the 0.547% tensile condition, and the thermal degradation of the HEMT was also obviously suppressed under compressive straining. The corresponding electrical performance changes and energy diagrams systematically illustrate the intrinsic mechanism. This work not only provides in-depth understanding of the piezotronic effect in tuning 2DEG and phonon properties in GaN HEMTs, but also demonstrates a low-cost method to optimize its electronic and thermal properties.

**Supplementary Information:**

The online version contains supplementary material available at (10.1007/s40820-021-00589-4).

## Highlights

- A large size (> 2 cm^2^) nitride membrane with High-electron-mobility transistor (HEMTs) arrays was successfully separated from sapphire substrate onto flexible substrate by an electrochemical lift-off technique.
- Without adding extra cost, the piezotronic effect is utilized to optimize the electric transport and thermal conductivity properties of the HEMTs by modulating the physical properties of the 2DEG and phonons.
- This study aims to open up a new way to fabricate high-performance GaN-based HEMTs and expand practical applications in flexible electronics.

**Supplementary Information:**

The online version contains supplementary material available at (10.1007/s40820-021-00589-4).

## Graphical abstract

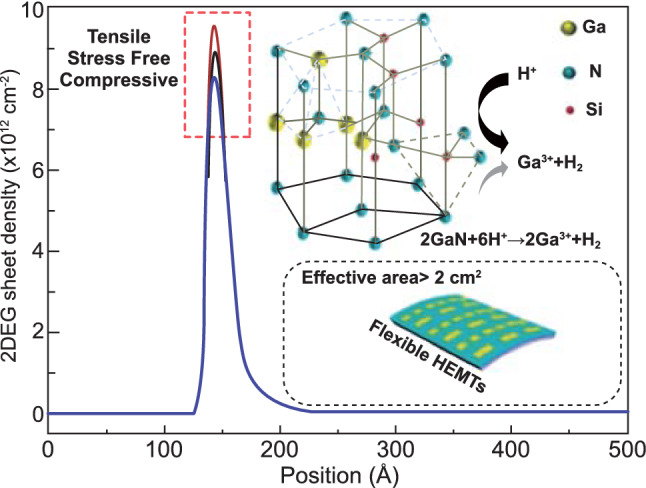

## Introduction

Owing to their high breakdown field strength, superior electron saturation drift speed, and excellent thermal conductance, nitride semiconductors have been widely used in various electronic/optoelectronic devices, such as light-emitting diodes (LEDs) [1, 2], photosensors [3–5], and high electron mobility transistors (HEMTs) [6, 7]. Among them, AlGaN/AlN/GaN heterostructure-based HEMTs take advantage of the high-density and high-mobility two-dimensional electron gas (2DEG) at the heterojunction interface, which has received much attention and shows extensive application prospects for radio-frequency (RF) devices and wireless transmission modules in communication. Future systems for HEMTs in intelligent devices, wireless communication and wearable devices have generated a demand for integration into diverse applications, circuits, platforms, and geometries. To more effectively accomplish this, the entire device will require a small footprint, possess flexural properties enable wireless systems to be placed onto nonplanar platforms or surfaces, and improve overall mechanical reliability. Significant efforts of flexible HEMTs have been made in strainable radio-frequency device [8], strain-controlled power devices (SPDs) [9], etc. However, the lattice tolerance and high growth temperature (> 1000 °C) [10] restrict their epitaxial growth mainly on rigid sapphire, SiC and/or Si by metal–organic chemical vapor deposition (MOCVD). In addition, the maximum potential of the HEMT is also limited by the high working temperature induced by near-junction Joule heating and insufficient rate of heat removal from the active device regions to the substrate, which significantly reduces the output performance and reliability [11, 12]. Mitigation of the thermal-induced degradation of HEMTs is strongly necessary. Therefore, the focus of implementing bendable HEMTs is to exfoliate from the original growth substrate and transfer to flexible substrates with high thermal conductivity. In addition, flexible substrates provide different functions such as flexible structures, stretchable structures, and curved conformal installations, which induce complex stress distribution to greatly affect the electrical characteristics [13]. Some transfer techniques have been reported and are shown in Table 1, such as the laser lift-off technique [14, 15], chemical lift-off (CLO) [16], mechanical lapping and etching of silicon-based HEMTs [17], and introducing an atomic-thickness release layer [18, 19], which increases the cost and risk due to the involvement of bulky and expensive equipment and/or strong acid. In addition, conductivity-selective electrochemical (EC) etching by preparing pre-holes on the epitaxial wafer layer [20, 21], inevitably destroys the effective area and integrity of NMs and largely limits the subsequent applications. Thus, it is necessary to develop a cost-effective and high-efficiency method for fabricating large bendable HEMTs.

**Table 1:** Previous reported transfer methods on GaN based nanomembranes (NWs)

Data sources	Methods	Process	Problem
[14, 15]	Laser lift-off (LLO)	GaN → Ga + N_2_	Uneven and whiskerlike micropole
[16]	Chemical lift-off (CLO)	HF/HNO_3_/CH_3_COOH	Harsh acid treatment; Hard to control
[17]	Mechanical lapping and etching	Alumina powder and abrasive disk and HF/HNO_3_/CH3COOH	Harsh acid treatment; Complicated process
[18, 19]	Mechanical release	Introducing atomic-thickness release layer (h-BN)	Unable to completely peel off the h-BN layer
[20, 21]	Electrochemical (EC) etching	Introducing sacrifcial layer and 50–100 μm holes on epitaxial wafer layer	Destroys the effective area and integrity of NMs

The piezotronic effect, a bridge connecting the piezoelectric property and non-centrosymmetrical semiconductors has been applied to optimize the performance of various nanodevices and demonstrate many novel applications [22, 23]. External-strain applied along the polar direction of noncentrosymmetrical semiconductors can generate piezoelectric polarization charges (piezo-charges) at the homo-/hetero-junction interfaces. These piezo-charges could modify the energy band and/or the local barrier height and ultimately affect the carrier transport. As mentioned above, the AlGaN/AlN/GaN heterostructures have strong spontaneous polarization and piezoelectric polarization, which results in the generation of net static charges and 2DEG at the interface. Under external stress conditions, the external strain-induced piezo-potential ($P_{{{\text{piezo}}}}$) would couple with the intrinsic spontaneous polarization ($P_{{{\text{sp}}}}$) and lattice-mismatch-induced piezoelectric polarization ($P_{{{\text{lm}}}}$). The coupling between these polarizations would fundamentally modify the energy band and change the charge density at the local heterojunction, and thus affect the performance of the HEMTs.

In this work, an electrochemical lift-off (ECLO) technique was adopted to peel AlGaN/AlN/GaN HEMT arrays from a sapphire substrate. A large nitride membrane with HEMT arrays was successfully separated from the sapphire substrate and transferred onto a polyethylene terephthalate (PET) flexible substrate. The analysis of the crystal quality indicates that the transferred membrane exhibited a decreased dislocation density. Moreover, the flexible devices showed a high saturation current of 105.67 mA mm^−1^ under zero gate voltage and excellent transconductance of 27.17 mS mm^−1^ at *V*_ds_ = 10 V. The piezotronic effect study indicated that the saturation current increased by 3.15% under the 0.547% tensile strain condition. In addition, the effects of external strain on the phonon properties and thermal conductivity of the flexible AlGaN/AlN/GaN HEMTs were systematically investigated. Under the compressive state, the saturation current attenuation of the device caused by thermal degradation was significantly mitigated. This study illustrates the intrinsic mechanisms of a 2DEG and thermal conductivity modulated by the piezotronic effect and endeavors to open up new ways to expand the practical applications of GaN-based HEMTs in flexible electronics.

## Experimental Section

### Synthesizing AlGaN/AlN/GaN Heterostructure Membrane

The multilayer epi-structure was fabricated by MOCVD. The trimethylgallium (TMGa), trimethylaluminum (TMAl), trimethylindium (TMIn), and ammonia (NH_3_) were used as Ga, Al, and N sources, respectively. N_2_ and H_2_ were used as carrier gases in the growth process. First grown on the sapphire substrate was a u-GaN (unintentional doped) layer, followed by a lightly doped n-type GaN (Si doping concentration of 5 × 10^18^ cm^−3^, 500 nm in thickness) layer, to improve the tangential current flow. After depositing another u-GaN (900 nm) layer which protects the n-GaN was a sacrificial layer (n^+^-GaN, *n* = 1.0 × 10^19^ cm^−3^, thickness of 1500 nm). The uppermost structure applied was an AlGaN/AlN/GaN heterojunction with a total thickness of 931.5 nm (Al_0.3_Ga_0.7_N layer of 30 nm, AlN layer of 1.5 nm; u-GaN of 900 nm). The detailed growth conditions are described in the following. GaN was synthesized at 1050 °C and 400 mbar in hydrogen for 1680 s using TMG (22 μmol min^−1^) and NH_3_ (67 mmol min^−1^). Afterward, an ultrathin AlN interlayer was deposited at 1100 °C for 8 s using TMAl (5.3 μmol min^−1^) and NH_3_ (90 mmol min^−1^). Finally Al_0.3_Ga_0.7_N layer was grown at 1200 °C for 50 s using TMGa (12.3 μmol min^−1^), TMGa (4.6 μmol min^−1^), and NH_3_ (110 mmol min^−1^). Before the device was prepared, the epitaxial wafer was cleaned with acetone, isopropanol, and deionized water, in that order. Subsequently inductively coupled plasma (ICP) was used to perform dry etching to form isolation regions, and finally photolithography was performed to form electrode patterns. The corresponding electrodes were fabricated by electron beam evaporation. The source and drain electrodes required ohmic contact; thus, four layers of metal Ti/Al/Ni/Au (20/130/50/100 nm) were deposited and then rapidly annealed in a nitrogen atmosphere at 850 °C for 30 s. As gate electrode required Schottky contact, electron beam evaporation was further used to deposit Ni/Au (30/300 nm). The gate length was 2 μm, the gate width was 60 μm, and the source–gate and drain–gate spacing were 3 μm and 10 μm, respectively.

### External Strain Calculations

External strains along the *c*-axis were applied to the AlGaN/AlN/GaN heterostructure membrane by locking the device between a one-dimensional displacement stage and a steady rest as shown in Fig. S6. By rotating the knob, a compressive or tensile strain was applied to the devices. For facilitate discussion, the bending strain was converted to the normal strain *ε* [24].

### Materials Characterization

The detailed microscopic structures of the epi-structures and the AlGaN/AlN/GaN heterostructure membrane were characterized by optical microscopy (LEICA DM500), scanning electron microscopy (SEM, ZEISS Ultra 55), transmission electron microscopy (TEM, JEM-2100HR) with selected-area electron diffraction (SAED), high-resolution HRTEM (HRTEM, JEM-1400 PLUS), Raman spectrometry (invia), Raman spectrometry (invia), high-resolution X-ray diffractometry (HRXRD, RIGAKU Smartlab 9 kW), and a Source/Measure Unit (Keysight B2902A) combined with a probe station.

## Results and Discussion

### Device Structure and Basic Characteristics

**Fig. 1:**
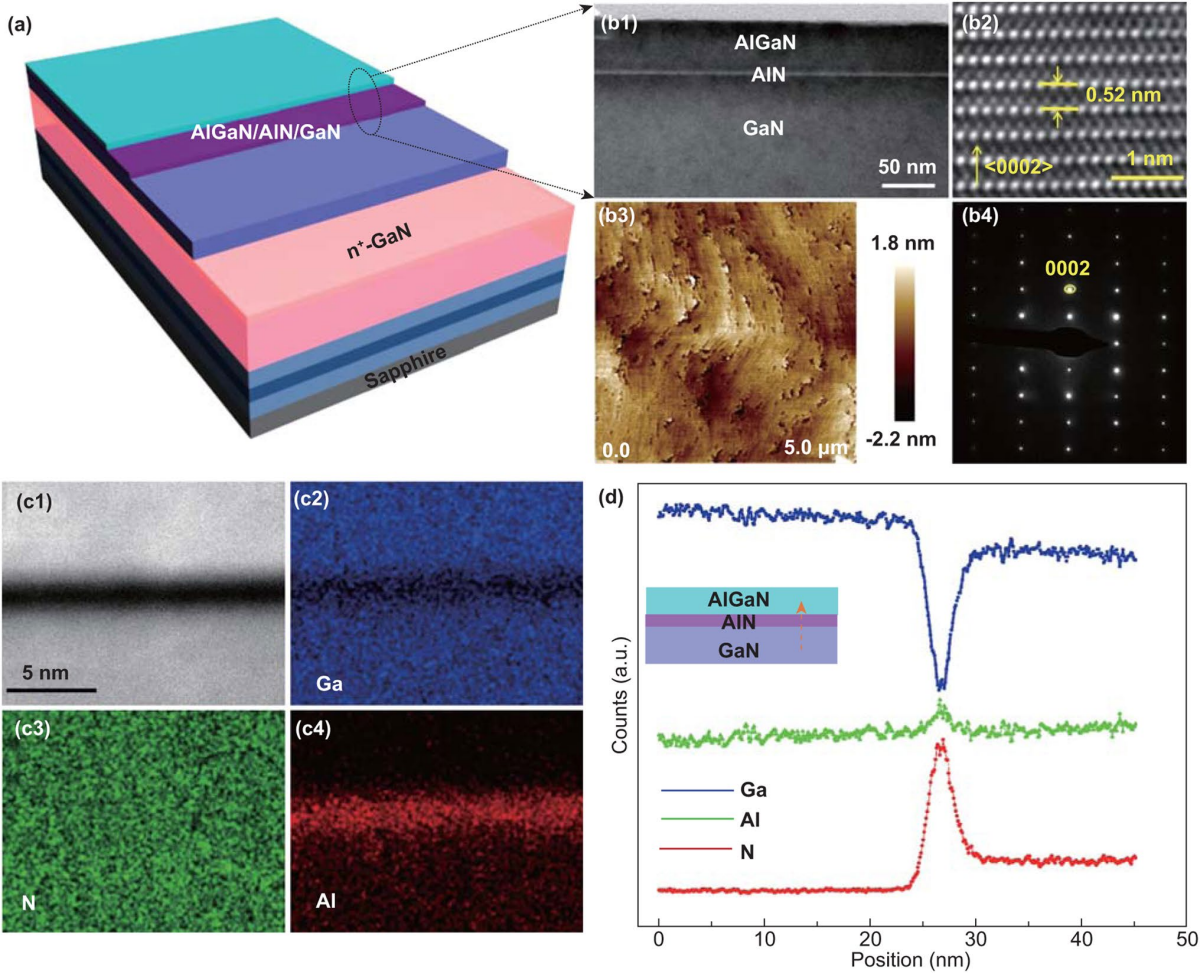
Structural characterization of AlGaN/AlN/GaN heterostructure membrane. **a** Schematic diagram of the heterostructure membrane on sapphire. **b1** The scanning transmission electron microscopy (STEM) image of AlGaN/AlN/GaN interface, the scale bar is 50 nm. **b2** The high-resolution transmission electron microscope (HRTEM) image. **b3** AFM image of heterostructure membrane on sapphire. **b4** The selected area electron diffraction (SAED) pattern taken from the same area of HRTEM. **c1** HAADF-STEM image collected from heterostructure membrane. **c2**–**c4** STEM energy-dispersive X-ray spectroscopy (EDX) elemental image of the AlGaN/AlN/GaN heterostructure. **d** The EDX line profiles for Ga (blue line), Al (green line), and N (red line) elements

Using MOCVD, the epitaxial structure was fabricated as shown in Fig. 1a. Details about the epitaxial multilayers were presented in Experimental Section. A specifically designed sacrificial layer (highly conductive GaN) was inserted between the AlGaN/AlN/GaN heterostructure and the GaN nuclear layer grown on sapphire, which was selectively etched in the ECLO process. Figure 1b1 shows a typical scanning transmission electron microscopy (STEM) image of an AlGaN/AlN/GaN interface, in which the white contrast line reveals the existence of an AlN ultrathin layer (1.5 nm). An HRTEM image of GaN is shown in Fig. 1b2; the interplanar spacing in the (0002) direction is 0.52 nm, which is consistent with bulk GaN [25]. The surface of the heterostructure membrane exhibits an atomically flat surface morphology under atomic force microscopy (AFM) (Fig. 1b3) with a root-mean-square roughness of less than 0.542 nm determined from an area of 5 × 5 μm^2^. In addition, the SAED pattern (Fig. 1b4) demonstrates that the epitaxial growth direction is along the < 0002 > *c*-axis. These morphological characterizations indicate that the GaN barrier underneath the ultrathin AlN layer is in a relaxed state and AlGaN/AlN/GaN heterostructure has outstanding epitaxial quality. The high-angle annular dark-field STEM (HAADF-STEM) image clearly shows the sandwich structure in Fig. 1c1 resulting from the insertion of AlN, and the corresponding spatial distributions of Ga, Al, and N elements are shown in Fig. 1c2–c4, which are the STEM energy-dispersive X-ray spectroscopy (EDX) elemental mapping of AlGaN/AlN/GaN heterostructure membrane. Along the direction from GaN to AlGaN, the results of EDX line-scan mapping were extracted and plotted in Fig. 1d. The sudden decrease/increase in the Ga/Al element further proves the existence of an ultrathin AlN layer and abrupt heterojunction interfaces.

**Fig. 2:**
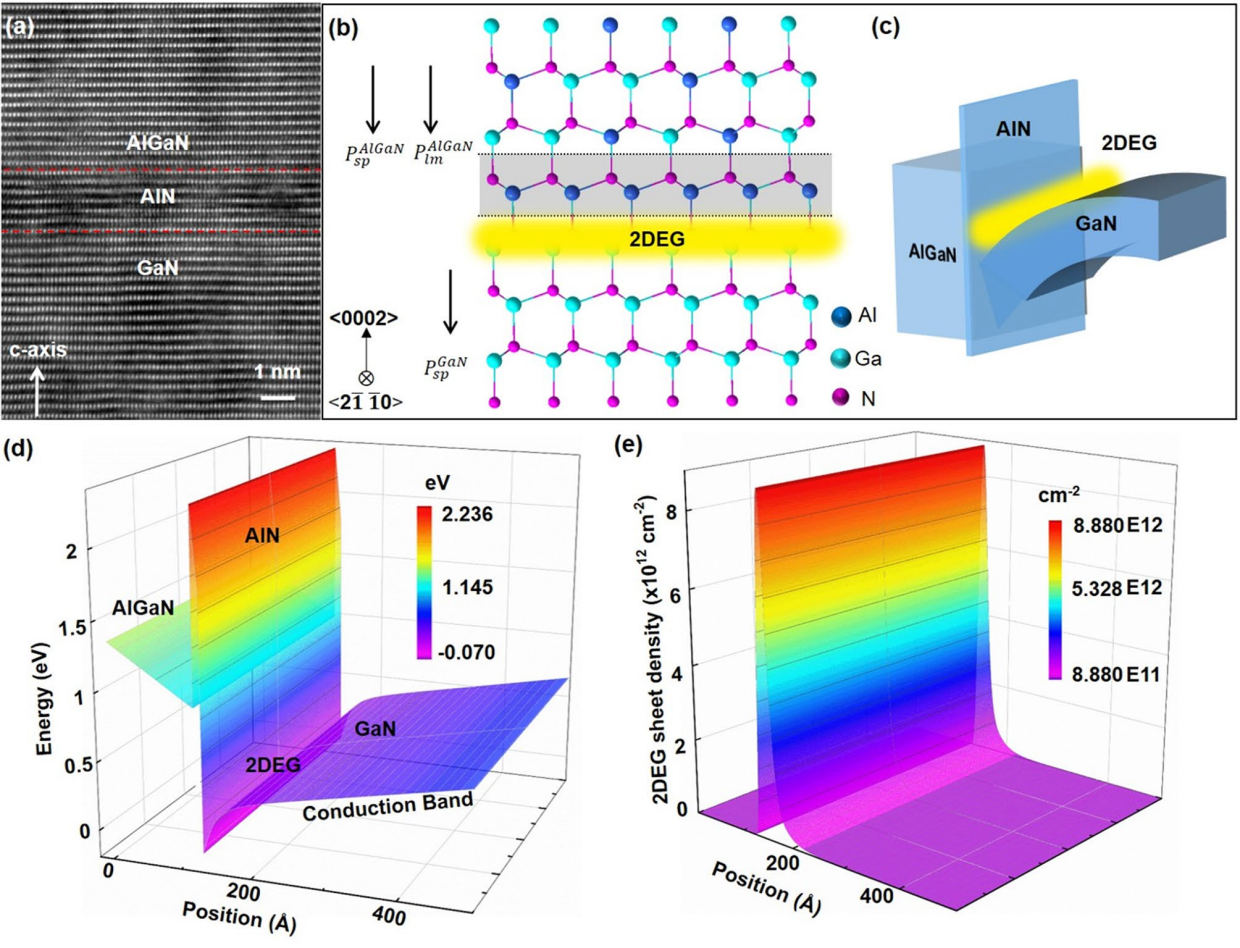
Atomic characterization of AlGaN/AlN/GaN heterostructure membrane. **a** The STEM image of heterostructure membrane. The scale bar is 3 nm. **b** Corresponding atomic structure along the c-axis. **c** The diagram of conduction band profile and 2DEG. **d** The calculated conduction band and valence band. **e** The calculated 2DEG sheet density in AlGaN/AlN/GaN heterostructure membrane

Figure 2 presents a detailed interfacial analysis of the internal polarizations, energy band and the induced 2DEG at the local AlGaN/AlN/GaN heterojunction. The lattice-resolved HAADF-STEM image (Fig. 2a) indicates the superior crystal quality and that the thickness of AlN is 1.5 nm. The AlN layer not only provides a barrier to strengthen the locality of the 2DEG in triangular potential wells, but also reduces 2DEG scattering by AlGaN. Figure 2b, c shows the atomic model and schematic band structure of the AlGaN/AlN/GaN heterojunction. Owing to the asymmetric lattice structure of the wurtzite materials, spontaneous polarization in AlGaN and GaN layer was formed, denoted as $P_{{{\text{sp}}}}^{{{\text{AlGaN}}}}$ and $p_{{{\text{sp}}}}^{{{\text{GaN}}}}$. In addition, piezoelectric polarization exists within the Al_*x*_Ga_1−*x*_N/GaN heterojunction induced by lattice mismatch, denoted as $p_{{{\text{lm}}}}^{{{\text{AlGaN}}}}$. The polarizations result in band bending at the heterojunction interface and form a triangle-shaped quantum potential well which constrains the high-density 2DEG (Fig. 2c, d). The existence of the 2DEG is confirmed by theoretical calculations and is indicated in Fig. 2e. This result confirms the high electron density of 8.8 × 10^12^ cm^−2^ is locating on the GaN side of the heterojunction.

**Fig. 3:**
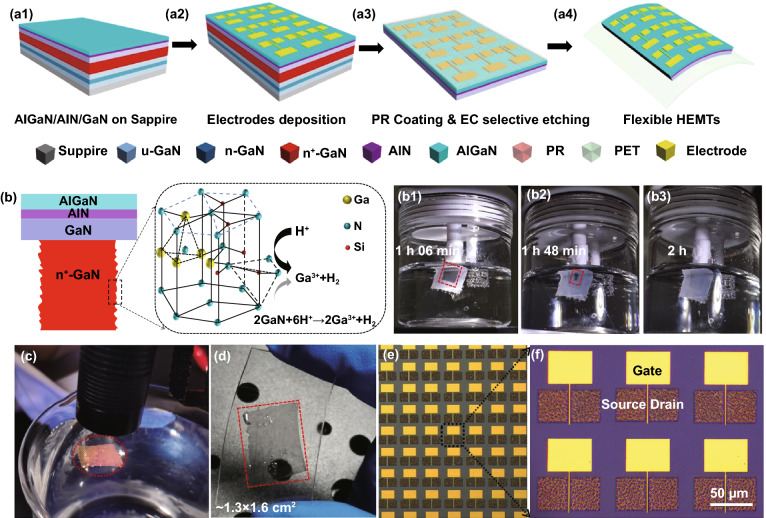
The HEMTs debonding and transition processes. **a1** Epitaxial layers on sapphire. **a2** The process of depositing electrodes. **a3** After coating photoresist, the selective EC eaching processes to lift-off sapphire. **a4** Transferring flexible HEMTs on PET substrate. **b** The process of lateral undercut eaching sacrificial layers n^+^-GaN and the etching mechanisms. **c**, **d** The optical images of successfully transferred large-area (~ 1.3 × 1.6cm^2^) and non-destructive HEMTs. **e**, **f** The microphotograph of HEMTs arrays

In order to acquire freestanding AlGaN/AlN/GaN HEMTs, ECLO was adopted. The advantages of ECLO are that it can be qualitatively selected based on differences in conductivity [26]. Thus a high doping concentration difference is significant for the ECLO. The n^+^-GaN, a sacrificial layer (red, Fig. 3a1) with doping concentration of 1.0 × 10^19^ cm^−3^, was buried under the AlGaN/AlN/GaN heterojunction in advance. The HEMT debonding and transition processes are revealed in Fig. 3a1–a4. First the electrodes were deposited (Fig. 3a2), and then a thick layer of photoresist (PR) was coated for ECLO (Fig. 3a3). Here, the PR has two roles, (1) protect the uppermost layers during ECLO; and (2) provide a mechanical support function, which is conducive to peel out a large area of complete flexible HEMTs. Next, EC etching was implemented. The AlGaN/AlN/GaN sample (with silver paste) as the anode was placed in 0.3 mol L^−1^ oxalic acid and a platinum sheet was used as the cathode. A constant voltage of 20 V was suitable for the EC etching. The etching process of the n^+^-GaN sacrificial layers and the formation of the freestanding AlGaN/AlN/GaN heterostructure membrane are reflected in the bright section (Fig. 3b1–b3). The etching mechanisms (Fig. 3b) can be explained by the following equation:

1 $$2{\text{GaN}} + 6{\text{h}}^{ + } \to 2{\text{Ga}}^{3 + } + {\text{N}}_{2}$$

After the etching, the HEMTs were still slightly attached to the grown substrate owing to van der Waals bonding. Therefore, we immersed the sample into DI water with a high surface energy to achieve the separation of the HEMTs. The completely released HEMTs was transferred onto PET flexible substrate. The presence of PR facilitated the transmission of strain from the PET to the HEMTs, potentially an end to curling and loosening. Finally, the PR layer was removed by acetone solution (Fig. 3a4). Figure 3c, d shows that a large area (~ 1.3 × 1.6 cm^2^) HEMTs arrays on the surface were successfully transferred from the sapphire substrate to the PET flexible substrate. Figure 3e–f are microphotographs of the flexible HEMTs showing the gate and source/drain contact, from which we can determine the flatness of electrodes and the complete array structure.

**Fig. 4:**
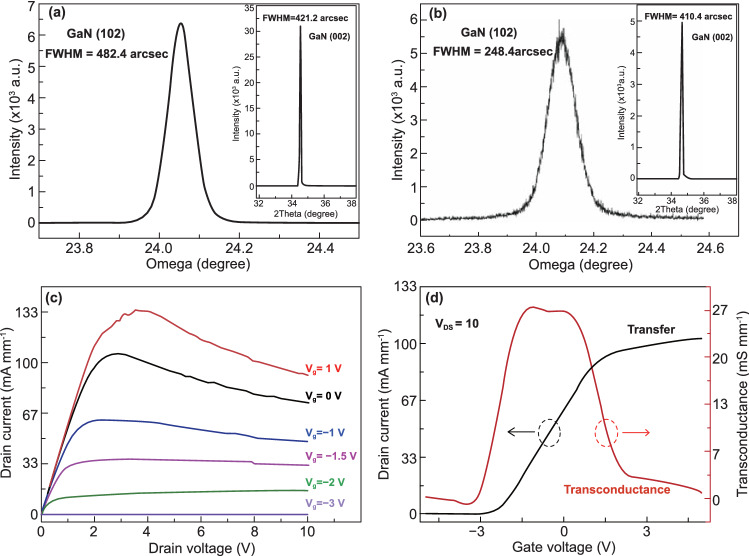
The crystal quality and electrical properties of flexible HEMTs. **a** The full width at half maximum (FWHM) of the GaN material measured by HRXRD of the AlGaN/AlN/GaN HEMTs on sapphire, and **b** on flexible PET. **c** Output curves under different gate voltages and **d** transfer characteristics of flexible HEMTs

The crystal quality of the buffer layer has a significant impact on all aspects performance of the HEMT performance. HRXRD testing was used to determine the dislocation density and crystalline quality of the AlGaN/AlN/GaN HEMTs before and after ECLO (Fig. 4a, b). The principal diffraction peaks are at 24.0°, 34.5°, corresponding to GaN (102) and GaN (002). The full width at half maximum (FWHM) of the (002) direction swing curve of the GaN on sapphire was 421.2 arcsec, and after being transferred to the flexible PET substrate, the FWHM decreased to 410.4 arcsec, while the FWHM of GaN (102) on sapphire and PET was 482.4 arcsec and 248.4 arcsec, respectively. The FWHM of the GaN (002) plane rocking curve can reflect the density of screw dislocations, while FWHM of the GaN (102) plane is related to the density of edge dislocations. The formula for calculating dislocation density is as follows [27]:

2 $$D = \frac{{\beta^{2} }}{{4.36b^{2} }}$$

where *D* is the screw or edge dislocation density, β is the FWHM value, and *b* is the Burger’s vector (*b*_screw_ = 0.5185 nm; *b*_edge_ = 0.3189 nm). The screw dislocation density can be calculated as 3.56 × 10^8^ cm^−2^ on the sapphire substrate and 3.38 × 10^8^ cm^−2^ on the PET. Similarly, the edge dislocation density is 3.27 × 10^8^ cm^−2^ on the PET. With the existence of sacrificial layers, some dislocations were introduced. As we can see, the dislocation density in the layer has a significant decline after stripping the sacrificial layers, which indicates that the contribution of sacrificial layers is excluded.

Due to the difference in lattice constants between GaN and the sapphire substrate, there should be residual stress in the epitaxial layer, which is seen in Fig. S1. In particular, the process of transferring an inflexible substrate to PET will have an effect on residual stress. Since the *E*_2_ (high) phonon vibration in the Raman spectra is sensitive only to internal residual stress [28], we estimate the change by observing the shift of *E*_2_ (high). The Raman spectra are shown in Fig. S2. For the as-grown AlGaN/AlN/GaN on sapphire, the *E*_2_ (high) peak is 570 cm^−1^ which is consistent with the standard *E*_2_ (high) peak of wurtzite-structured GaN. The *E*_2_ (high) peak shifted to 567 cm^–1^ for the flexible HEMTs. The formula for calculating the internal residual stress is as follows [29, 30]:

3 $$\Delta \sigma_{{\text{a}}} = \frac{{\Delta \omega_{{E_{2} }} }}{{k_{{{\text{Raman}},{\text{a}}}} }}$$

where $\sigma_{{\text{a}}}$ is the residual stress, $\Delta \omega_{{E_{2} }}$ is the difference of the *E*_2_ (high) peak, and $k_{{\text{Raman,a}}}$ = 4.2 cm^−1^ GPa^−1^. Because the lattice constant of the sapphire substrate is larger than that of GaN, the epitaxially grown GaN layer is subject to tensile stress in the horizontal direction and compressive stress in the vertical direction. The corresponding stress obtained by the calculation is 714.2 MPa. The red-shift of the Raman peak in the free-standing AlGaN/AlN/GaN heterostructure membrane is mainly attributed to the released residual compressive stress during stripping. These results indicate that the ECLO techniques can effectively release the compressive stress of the original structure and reduce the polarization at the interface.

The DC output current–voltage (*I*_ds_*–V*_ds_) and transfer properties are shown in Fig. 4c, d. More details of the measurement set-up and processes are presented in Figure S3 (Supporting Information). When the source-drain voltage is small, the curves have good linearity characteristics, and the increase in current is consistent with the change in the source-drain voltage. As the drain voltage increases further, the source-drain current reaching saturation which is attributed to the electron drift velocity reached the saturation velocity. However, in the saturation region at large positive gate voltages, a negative differential resistance is observed owing to the self-heating effect. The device reaches a maximum saturation current of 105.67 mA mm^−1^ at a gate voltage of 0 V. The transfer characteristics of the device were measured at source-drain bias of 10 V, where the HEMTs were completely pinched off with a negative gate of − 2.38 V (*V*_th_ =  − 2.38 V), which proves excellent electrical characteristics of the device (Fig. 4d). The Schottky contact of the gate is realized by the Ni/Au alloy, while, the gate is a switch that controls 2DEG in the channel. By differentiating the transfer characteristics, the relationship between the transconductance and gate voltage can be obtained (Fig. 4d). The maximum transconductance was 27.17 mS mm^−1^ at *V*_ds_ = 10 V. The transconductance of GaN HEMT have been compared with the previously reported GaN-based HEMTs [7, 9, 31, 32], and showed in Table 2. With the increase in the thickness of AlGaN barrier layer and Al composition, the sheet density of 2DEG is increasing, while the enhancement of electron scattering leads to the decrease in the 2DEG mobility, resulting in a relatively low transconductance. Further, the unintentionally doped GaN cap layer grown on AlGaN/AlN/GaN can reduce the leakage current and improve gate control performance [32]. Additionally, the electric measurement of the HEMTs before lift-off was also conducted (Fig. S4). Under different gate voltages (1 V,–1 V), the *I*_ds_*-V*_ds_ curves exhibit linear characteristics, at the same time, the gate control effect is inconspicuous. This result is attributed to the existence of the sacrificial layers which are also high conductivity layers and serve as another electron transport path (Fig. S5).

**Table 2:** Comparison of transconductance in GaN HEMTs

Data sources	Device structure	Gate length (μm)	*G*_m_ (mS mm^−1^)
[9]	Al_0.3_Ga_0.7_N(30 nm)/AlN/GaN	5	7.5 (Schottky-gate)
[31]	Al_*x*_Ga_1–*x*_N(20 nm)/AlN/GaN	3	29 (With SiO_2_ dielectric layer)
[7]	Al_0.3_Ga_0.7_N(20 nm)/AlN/GaN	–	40 (With Al_2_O_3_ layer)
[32]	Al_0.3_Ga_0.7_N(50 nm)/AlN/GaN	5	4 (Schottky-gate)
This work	Al_0.3_Ga_0.7_N(30 nm)/AlN/GaN	2	27 (Schottky-gate)

### Strain Engineering of AlGaN/AlN/GaN Heterostructure Membrane

**Fig. 5:**
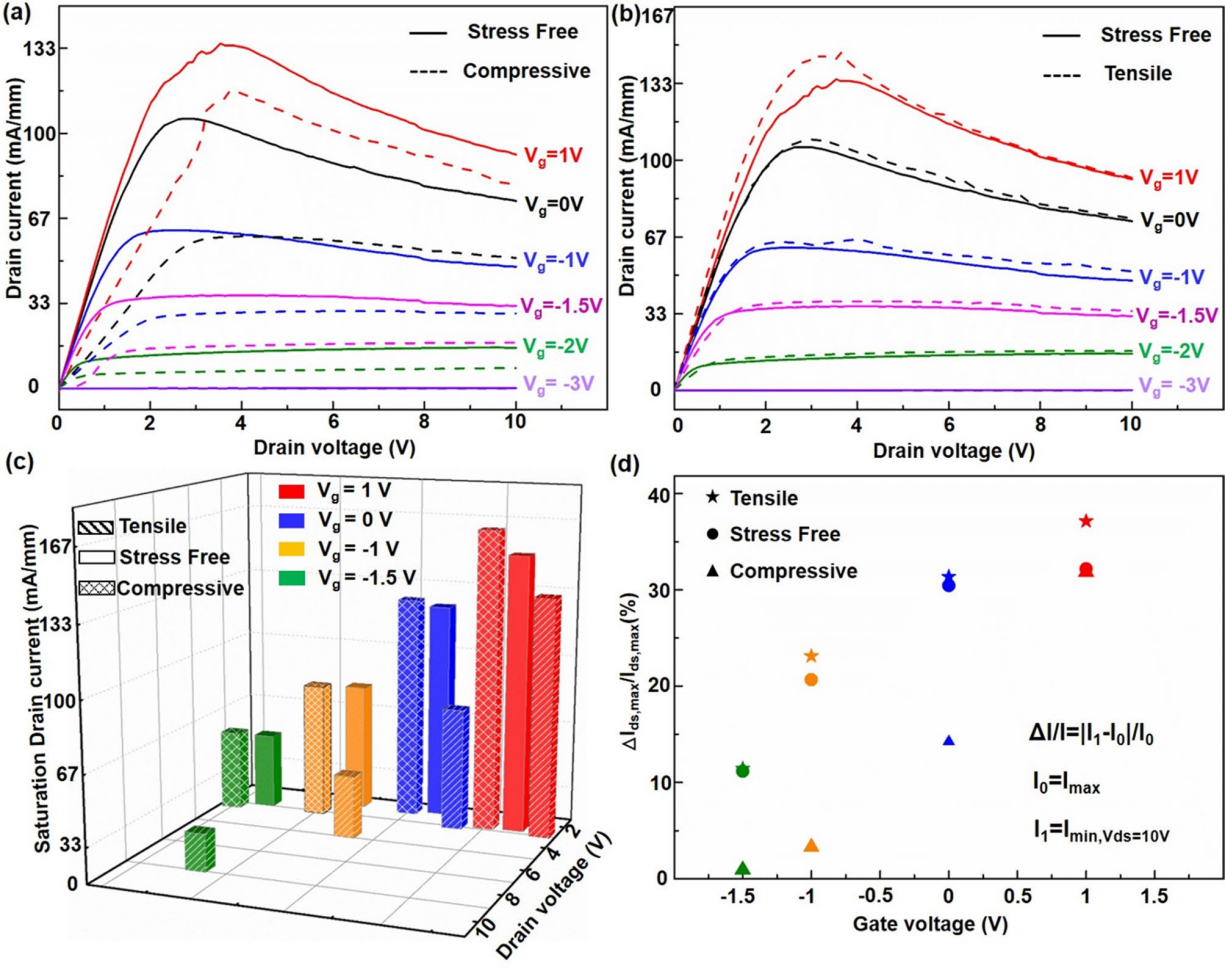
Piezotronic effect and investigate on saturation drain current attenuation. **a** *I–V* characteristics of HEMTs under strain free, compressive and **b** tensile strain condition. **c** The saturation drain current value and the corresponding peak position changes. **d** The corresponding relative changes (*ΔI*_ds,max_*/I*_ds,max_)

To further explore the intrinsic mechanism of the piezotronic effect on the fabricated HEMTs, strain engineering of the AlGaN/AlN/GaN heterostructure membrane was studied systematically under different strains. A schematic diagram of the experimental device and membrane is shown in Fig. S6. Details about applying and calculating strains on the devices are provided in the Experimental Section. The *I*_ds_*-V*_ds_ characteristics of HEMTs under − 0.547% compressive strain and 0.547% tensile strain are summarized in Fig. 5a, b, respectively. From figure, it is clear that the *I*_ds_ of the HEMTs decreased as compressive strain was applied, and increased with tensile strain, confirming that the 2DEG at the AlGaN/AlN/GaN heterostructure membrane is tuned by the piezotronic effect. Figure 5c plots the saturation drain current value and the corresponding peak position changes as a function of external strain and the influence of lattice thermal conductivity. It is conducted by calculating the relative changes of the saturation drain current *ΔI/I* = *(I*_1_*-I*_0_*)/I*_0_, where *I*_0_ = *I*_strain = 0.00%_; *I*_1_ = *I*_strain = − 0.547%_ or *I*_1_ = *I*_strain=0.547%_ as shown in Fig. 5c. More details can be found in Table S1 (Supporting Information). For the 0.547% tensile strain, the calculated *ΔI/I* increases by 3.15%, while for the − 0.574% compression strain, the calculated *ΔI/I* decreased by 43.53%. This clearly indicates that the output current of the HEMTs increases/decreases with the tensile/compression strain, especially significantly under compression strain. Further investigation of the piezotronic effect on the thermal conductivity of HEMTs was carried out by calculating the relative change of the saturation drain current attenuation *ΔI/I* =*|I*_1_*-I*_0_*|/I*_0_, where *I*_0_ = *I*_ds,Max_; *I*_1_ = *I*_min,Vds = 10v_ as shown in Fig. 5d. A significant decrease in *I*_ds_ with increasing *V*_ds_ and, negative differential resistance, is observed in the saturation region. At a gate voltage of 0 V, the calculated *ΔI/I* decreases by 31.35%, 30.44%, and 14.25% under the tensile/free/compression state, as shown in Table 3. The presence of negative differential resistance is commonly attributed to the self-heating effect [33]. It is obvious that the responses of thermal conductivity are different in the compression/tensile strain states. An improved thermal property will reduce the electron scattering. In principle, strain can influence the thermal conductivity by tuning the phonon properties based on the phonon Boltzmann transport equation [34]:

4 $$K_{L}^{\alpha \beta } = \frac{1}{{k_{B} T^{2}\Omega N}}\mathop \sum \limits_{q\nu } f_{o} \left( {f_{o} + 1} \right)\left( {\hbar \omega_{q\upsilon } } \right)^{2} \upsilon_{q\upsilon }^{\alpha } F_{q\upsilon }^{\beta }$$

where $f_{o}$ is the Bose–Einstein distribution function, $\omega_{q\upsilon }$ is the phonon angular frequency in wavevector q and polarization $\upsilon$, and $\upsilon$ is the phonon group velocity. *F* is the product of the phonon group velocity and converged relaxation time $\upsilon_{q\upsilon }^{\alpha } \tau_{q\upsilon }$, over distance, and can be simplified into $\upsilon_{q\upsilon }^{\alpha } \tau_{q\upsilon }^{0}$ under relaxation time approximation (RTA), where $\tau^{0}$ is the phonon relaxation time for the perturbation theory. Compressive strain increases the thermal conductivity as it increases the specific heat and phonon group velocity. At the same time the phonon free path and larger bandgap between the highest frequency phonons in the lower part and the lowest frequency phonons in the higher part will suppress the three-phonon scattering processes. This confirm that the thermal conductivity can be modulated by the piezotronic effect, and it increases under compressive strain and decreases under the tensile state. In practical terms, strain engineering on GaN-based flexible HEMTs is a positive method to simultaneously enhance performance and suppress thermal degradation.

**Table 3:** The relative change of saturation drain current attenuation

Δ*I*/*I* =|*I*_1_ − *I*_0_|/*I*_0_	Tensile state (%)	Strain free (%)	Compressive state (%)
*V*_g_ = 1 V	37.12	32.18	31.81
*V*_g_ = 0 V	31.35	30.44	14.25
*V*_g_ = − 1 V	20.66	23.11	3.30
*V*_g_ = − 1.5 V	11.16	11.42	0.93

### Physical Mechanisms

**Fig. 6:**
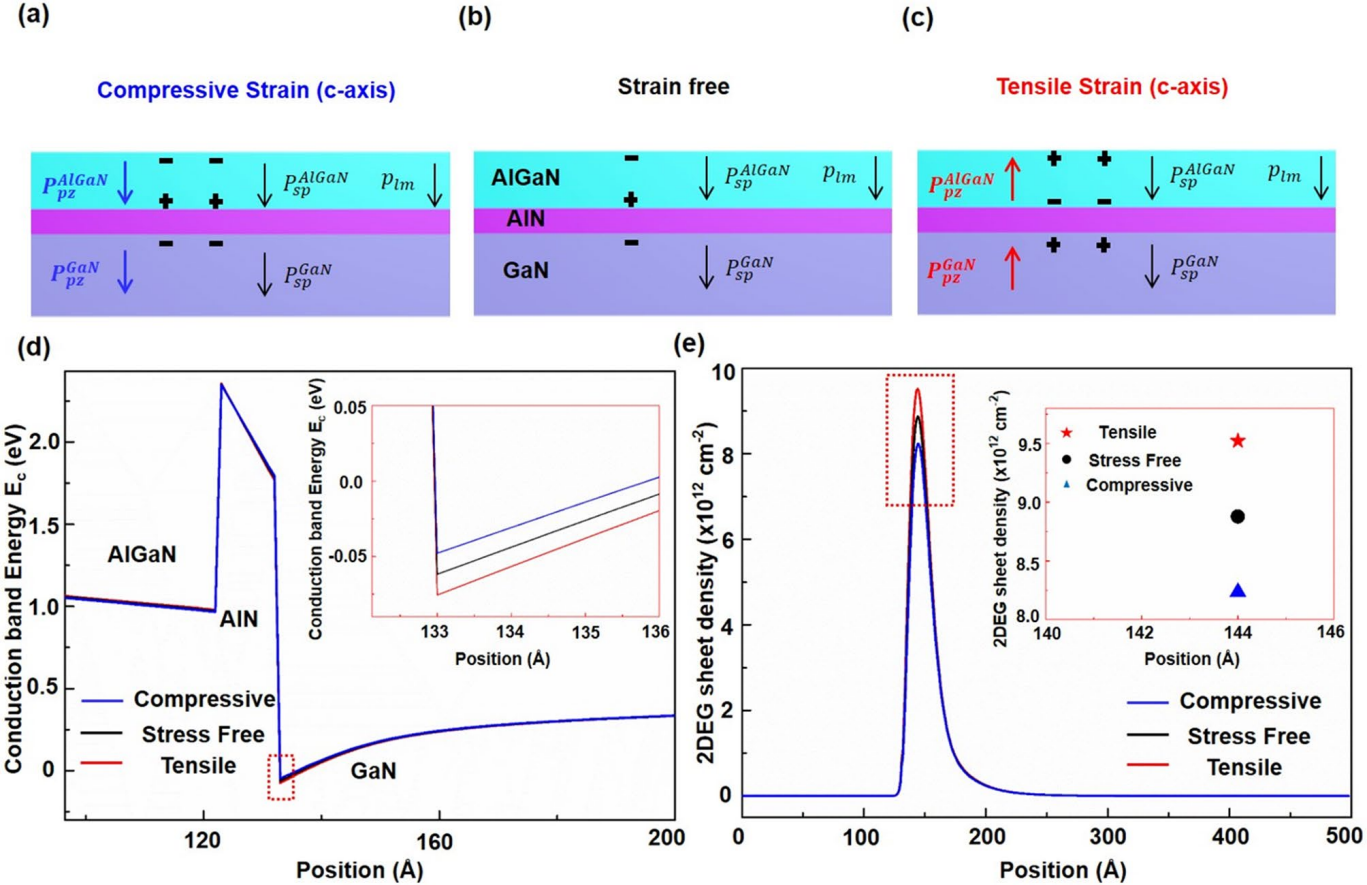
Working mechanism of the piezotronic effect and Self-consistent calculation of energy profiles and 2DEG sheet density. The coupling of piezotronics-induced polarization and intrinsic polarization (p_sp_;p_lm_), corresponding energy band profiles, and distribution of 2DEG in AlGaN/AlN/GaN HEMTs under **b** strain free, **a** compressive and **c** tensile strain states. **d** The conduction band energy profiles under compressive strain state (blue line); stress free state (black line); tensile strain state (red line). **e** The sheet density of 2DEG under under compressive strain state (blue line); stress free state (black line); tensile strain state (red line)

The energy band diagrams and 2DEG sheet density of the AlGaN/AlN/GaN heterostructure membrane along the *c*-axis are demonstrated in detail by the Poisson equation and self-consistency of the Schrödinger equation [35, 36], under various straining states to systematically explain the intrinsic mechanism of 2DEG modulated by piezoelectric effects. Under the strain-free state (Fig. 6b), the coexistence of larger energy band discontinuities,$P_{{{\text{sp}}}}^{{{\text{AlGaN}}}}$, $P_{{{\text{sp}}}}^{{{\text{GaN}}}}$ and $P_{{{\text{lm}}}}^{{{\text{AlGaN}}}}$, together contribute to band bending at the heterojunction interface and form a triangle-shaped quantum potential well. Bound positive charges are formed on the bottom surface of the AlGaN layer due to the existence of $P_{{{\text{sp}}}}^{{{\text{AlGaN}}}}$ and $P_{{{\text{lm}}}}^{{{\text{AlGaN}}}}$, while bound negative charges are formed on the upper surface of the GaN film due to $P_{{{\text{sp}}}}^{{{\text{GaN}}}}$. The net fixed charges at the heterojunction interface are always positive regardless of the stress condition, attracting free negative charges near the heterojunction interface; thus, most of the positive charges are compensated, which gives rise to the formation of the 2DEG in the potential well. Therefore, the net fixed charges are directly related to the density of the 2DEG, which in turn determines the electrical performance of the device. Under compressive strain along the c-axis (Fig. 6a), positive piezoelectric polarization charges (piezo-charges) are induced at the − *c*-plane, while negative piezo-charges are induced at + *c*-plane. The decreased net fixed charges release a certain amount of restricted free electrons, reducing the sheet density of the 2DEG. Meanwhile, the energy band of the AlGaN close to the AlGaN/AlN interface is bent downward under the action of positive piezo-charges, and the energy band of the GaN close to the AlN/GaN interface is bent upward under the action of negative piezo-charges. Thus, the potential well is “shallower” (Fig. 6d, blue line) confirming fewer electrons within, indicating that the 2DEG decreases as the electron transport of the HEMTs is weakened (Fig. 4a). In contrast, under tensile strain along the c-axis (Fig. 6c), negative piezoelectric polarization charges (piezo-charges) are induced at the − *c*- plane, while positive piezo-charges are induced at the + c-plane. The energy band of the AlGaN close to the AlGaN/AlN interface bends upward under the action of the negative piezo-charges, and the energy band of the GaN close to the AlN/GaN interface bends downward under the action of positive the piezo-charges. Thus, the potential well is “deeper” (Fig. 6d, red line) confirming more electrons within, as shown in Fig. 4b, which agrees with the enhanced output characteristics. Figure 6e shows the change in the 2DEG sheet density under different strain states and the maximum extraction diagram (Fig. 6e, inset), clearly showings that the 2DEG density increases with the tensile strain and decreases with the compressive strain and further demonstrating the piezotronic effect on the 2DEG.

## Conclusion

ELCO technology was utilized to prepare flexible GaN HEMT arrays with a size of > 2cm^2^. The piezotronic effect was then introduced to the AlGaN/AlN/GaN heterostructure membrane as an effective approach to modulate the physical properties of the 2DEG and phonon in the HEMTs, and thus control the electron transport process and thermal conductivity. Compared to existing traditional methods, the piezotronic effect has particular advantages, introducing external stress exhibits unique advantages of convenience, cost and reliability. By coupling the piezotronic effect into the device, the saturation current increased by 3.15% under the 0.547% tensile state, while the thermal conductivity of the HEMTs was enhanced monotonously and significantly under the compressive strain state. The piezotronic effect on the 2DEG, thermal conductivity and the coupling between the external strain-induced $P_{{{\text{piezo}}}}$ and the intrinsic $P_{{{\text{sp}}}}$ and $P_{{{\text{lm}}}}$ were demonstrated in detail to systematically explain the intrinsic mechanism. This study provides an in-depth understanding of the piezotronic-effect and endeavors to open new ways for thermal management, with practical applications in flexible electronics.

## Supplementary Information

Below is the link to the Supplementary Information.

Supplementary Information 1 (DOCX 1663 kb)
