# Supplementary material for: Epitaxial Lift-Off of Flexible GaN-Based HEMT Arrays with Performances Optimization by the Piezotronic Effect
Source: Nanomicro Lett. 2021 Feb 10;13:67. doi: 10.1007/s40820-021-00589-4 (PMC8187690; doi:10.1007/s40820-021-00589-4)
Supplement: Supplementary file 1 — Supplementary Information 1 (DOCX 1663 kb) [file 40820_2021_589_MOESM1_ESM.docx]

Supporting Information

**Epitaxtial lift-off for flexible arrayed GaN-based HEMT and its performances optimization by the piezotronic effect**

Xin Chen ^1, #^, Jianqi Dong ^1, #^, Chenguang He ^2^, Longfei He ^2^, Zhitao Chen ^2^, Shuti Li ^1^, Kang Zhang ^2, *^, Xingfu Wang ^1, *^, and Zhong Lin Wang ^3, 4, *^

^1^Laboratory of Nanophotonic Functional Materials and Devices, Institute of Semiconductor Science and Technology, South China Normal University, Guangzhou, China, 510631

^2^Institute of Semiconductor, Guangdong Academy of Sciences, Guangzhou, China, 510651

^3^Beijing Institute of Nanoenergy and Nanosystems, Chinese Academy of Sciences Beijing 100083, P. R. China

^4^School of Materials Science and Engineering Georgia Institute of Technology Atlanta 30332-0245, GA, USA

***Corresponding authors. E-mail address:

[zhong.wang@mse.gatech.edu](mailto:zhong.wang@mse.gatech.edu) (Prof. Zhong Lin Wang)

[xfwang@scnu.edu.cn](mailto:xfwang@scnu.edu.cn) (Prof. Xingfu Wang)

[kangzhang@gdisit.com](mailto:kangzhang@gdisit.com) (Dr. Kang Zhang)

^#^These authors contributed equally to this work.

**Supplementary Notes:**

***The calculation of intrinsic polarization in AlGaN/AlN/GaN heterostructure membrane:*** The intensity of spontaneous polarization in GaN is -0.034 C/m^2^, in AlN is -0.090 C/m^2^ which the direction is opposite to the +c-axis. The intensity of spontaneous polarization in AlGaN can be calculated from the formula [1]:

$$p_{sp}\left( {Al}_{x}{Ga}_{1-x}N \right)=\left[ -0.09x-0.034\left( 1-x \right)+0.019x\left( 1-x \right) \right] C/{m^{2}} \left( 1 \right)$$

Where x represents Al component.

In our work, Al component is 0.3, thus the intensity of spontaneous polarization in AlGaN is -0.0468 C/m^2^. The lattice constants of AlN and GaN are 0.3112 nm and 0.3189 nm, the former is about 2.4% smaller than the latter. Thus, when the Al_x_Ga_1-x_N epitaxially fabricated on the GaN crystal is less than the critical thickness, a strained heterojunction structure will be formed, the Al_x_Ga_1-x_N film undergoes tensile strain in the lateral direction and compressive strain in the longitudinal direction. Thus, the lattice-mismatch piezoelectric polarization in Al_x_Ga_1-x_N can be obtained from the formula [2]:

$$p_{pz}\left( {Al}_{x}{Ga}_{1-x}N/GaN \right)=\left[ -0.0525x+0.0282x\left( 1-x \right) \right]C/{m^{2}} \left( 2 \right)$$

Where x represents Al component.

The intensity of piezoelectric polarization in Al_x_Ga_1-x_N (x=0.3) is -0.0098 C/m^2^. Consider the combined effect of spontaneous polarization and piezoelectric polarizationin in AlGaN/GaN heterojunction, the total polarization intensity in AlGaN can be given from:

$$p_{{Al}_{x}{Ga}_{1-x}N}=p_{sp}\left( {Al}_{x}{Ga}_{1-x}N \right)+p_{pz}\left( {Al}_{x}{Ga}_{1-x}N/GaN \right)C/{m^{2}} \left( 3 \right)$$

And the calculation results is -0.0566 C/m^2^. At the Al_0.3_Ga_0.7_N/GaN heterojunction interface (AlN ultrathin layer is not considered), the net fixed charges can be estimated from:

$$p_{{Al}_{0.3}{Ga}_{0.7}N/GaN}^{Total}=p_{{Al}_{x}{Ga}_{1-x}N}+p_{GaN}^{sp} \left( 4 \right)$$

The net fixed charges at interface are 0.0226 C/m^2^, which are always positive regardless of the stress condition.

Figure S1


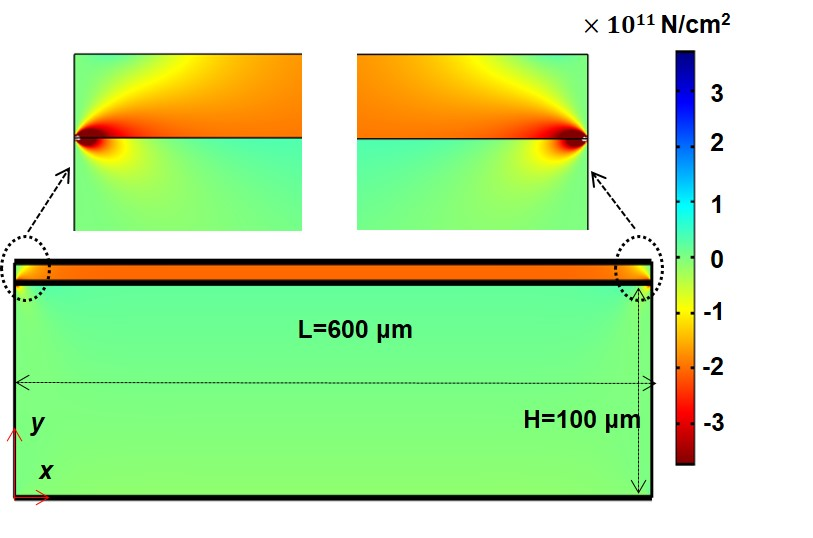


**Figure S1. The simulated stress distribution of AlGaN/AlN/GaN heterostructure membrane.** The model is the as-grown AlGaN/AlN/GaN heterostructure membrane. The partial enlarged detail is selected in the range y=100 to y=110 μm and x=600 μm.

Figure S2

**
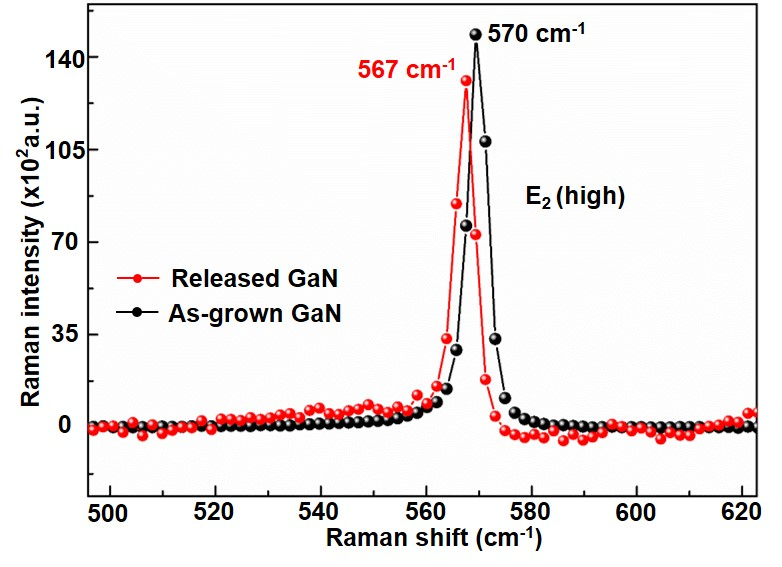
**

**Figure S2.** **Roman scattering measured from as grown as-grown AlGaN/AlN/GaN heterostructure membrane on sapphire (black line) and after (red line) releasing.**

Figure S3


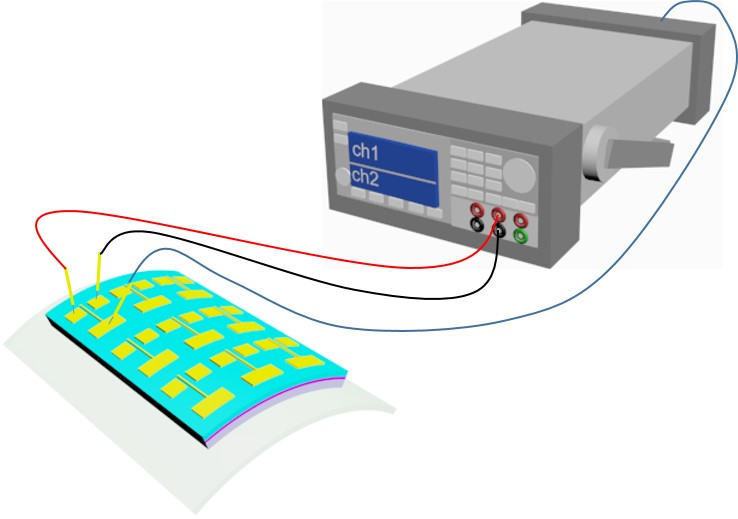


**Figure S3. The schematic diagram of Keysight B2902A Precision Source/Measure Unit combined with a probe station to measure DC *I_ds_-V_ds_*.**

Figure S4


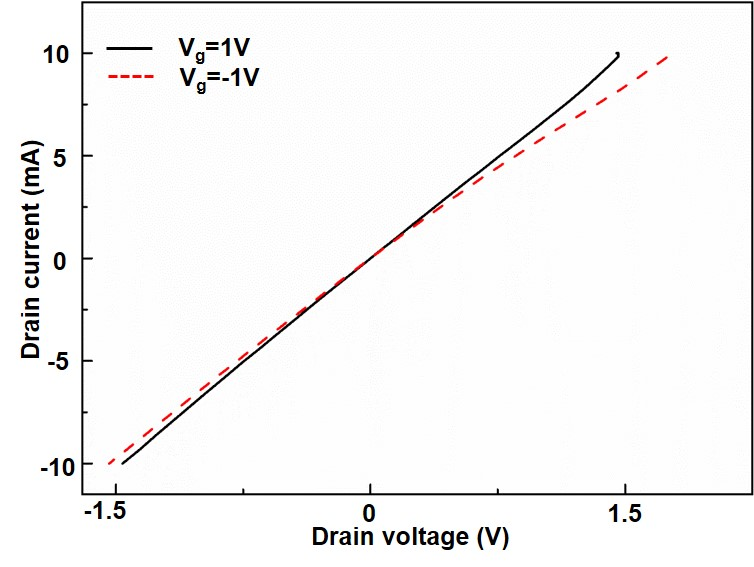


**Figure S4. The electrical characteristics measure of HEMTs on sapphire, with sacrificial layers.**

Figure S5

**
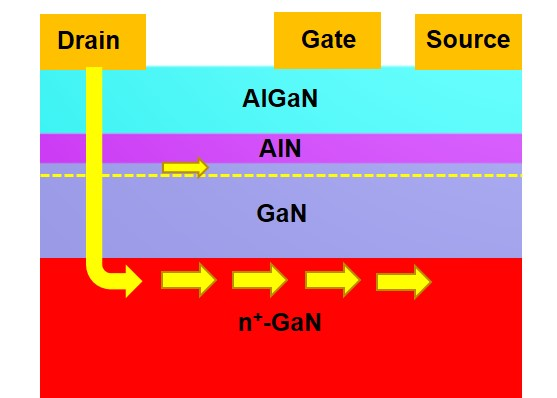
**

**Figure S5. The schematic diagram of electron transmission path with the existence of n^+^-GaN layer.**

Figure S6

**
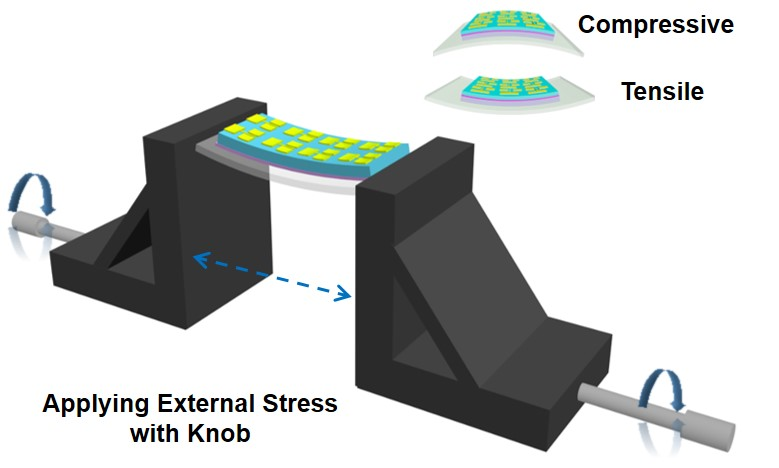
**

**Figure S6. The schematic diagram of experimental device and membrane for inducing external strain.**

**Table S1. The relative change of saturation drain current value and the corresponding peak position under different strain state.**

| (I_stress_-I_0.00_)/I_0.00_ | Compression strain | Tensile strain |
| --- | --- | --- |
| Vg=1V | -13.56% | 8.63% |
| Vg=0V | -43.53% | 3.15% |
| Vg=-1V | -51.08% | 5.38% |
| Vg=-1.5V | -50.68% | 6.39% |

**Supplemental References:**

1. O. Ambacher, J. Smart, J. R. Shealy, N. G. Weimann, K. Chu, M. Murphy, W. J. Schaff, L.F. Eastman, R. Dimitrov, L. Wittmer, M. Stutzmann, W. Rieger, J. Hilsenbeck, Two-dimensional electron gases induced by spontaneous and piezoelectric polarization charges in N- and Ga-face AlGaN/GaN heterostructures. J. Appl. Phys. **85**, 3222-3233 (1999).

<https://doi.org/10.1063/1.369664>

1. V. Fiorentini, F. Bernardini, O. Ambacher, Evidence for nonlinear macroscopic polarization in III–V nitride alloy heterostructures. Appl. Phys. Lett. **80**, 1204-1206 (2002).

<https://doi.org/10.1063/1.1448668>
